# Caesarean Section frequency in Nulliparous Women induced at 39 weeks versus conventional management: An open label random allocation study

**DOI:** 10.12669/pjms.40.8.9099

**Published:** 2024-09

**Authors:** Erum Majid, Bader Faiyaz Zuberi, Kanwal Gul, Hira Jam, Gul Haseena

**Affiliations:** 1 Erum Majid, FCPS Associate Professor Department of Obstetrics/Gynaecology, Jinnah Postgraduate Medical Center (JPMC), Karachi, Pakistan; 2 Bader Faiyaz Zuberi, FCPS Meritorious Professor, OMI Hospital, Karachi, Pakistan; 3 Kanwal Gul, MBBS, RMO Hira Jam, MBBS Post-graduate Resident Department of Obstetrics/Gynaecology, Jinnah Postgraduate Medical Center (JPMC), Karachi, Pakistan; 4 Hira Jam, MBBS Post-graduate Resident Department of Obstetrics/Gynaecology, Jinnah Postgraduate Medical Center (JPMC), Karachi, Pakistan; 5 Gul Haseena Post-graduate Resident Department of Obstetrics/Gynaecology, Jinnah Postgraduate Medical Center (JPMC), Karachi, Pakistan

**Keywords:** Induction of labour, Caesarean section, Maternal outcome, Foetal outcome

## Abstract

**Objective:**

To compare frequency of caesarean section in singleton primary-para women induced at 39 week and its comparison with conventional management. The other objective was comparison of perinatal and neonatal outcomes.

**Method:**

Open random allocation study was conducted at Gynae/Obst Department JPMC during period from 1^st^ June 2022 to 30^th^ September 2023. Primiparous women with singleton pregnancy without risk factors with gestational age 38 weeks 0 days to 38 weeks six days attending the anti-natal clinic offered to participate after consent. Non-probability convenience sampling method was used for induction. Randomization was done using random number table into one of the two groups, Group-A in which induction was done at 39 weeks while in Group-B induction was done conventionally. Mean age ±SD, gestational age and delivery time was calculated and compared by Student’s t test. Frequency of CS, perinatal and neonatal outcomes was compared by χ^2^ test.

**Results:**

Eighty-two women were inducted in Group-A and eighty-five in Group-B. The mean delivery time in Group-A was significantly more at 8.12±2.77 hours while in Group-B was 7.0±2.62 hours (*p* = .005). Frequency of CS between two groups was not statistically significant, it was 5 (6.1%) in Group-A and 2 (2.4%) in Group-B (*p* = 0.412). No significant difference in frequency of NICU admission was seen, in Group-A 8.54% babies were admitted to NICU while in Group-B 16.47% were admitted to NICU (*p* = 0.122).

**Conclusion:**

No significant difference was observed in frequencies of CS, Foetal, Neonatal, and Maternal outcomes.

## INTRODUCTION

Inducing Labour between 38 to 39 weeks has recently decreased the caesarean delivery rate and perinatal mortality.^1^-^3^ The rising caesarean section (CS) worldwide has raised many questions, due to the associated morbidity and mortality. Induction of Labour (IoL) at this gestation has proven to decrease rates of stillbirth, preeclampsia, macrocosmic babies and decrease pelvic floor injuries.^4^,^5^ This fact is changing the older trend of expectant management in pregnant women till 41 weeks. Conversely, there are studies that show the chances of operative delivery are more with induction of labor (IoL). Conversely, induction of labor may result in category II and category III foetal heart rate tracing with hyperstimulation and may result in a higher CS rate, especially in women with unfavorable cervixes.^6^

WHO guidelines regarding induction have cut off 40 to 41 weeks for intervention.^7^,^8^ If waiting till 40 weeks carries the risk of perinatal and maternal risks, then it’s time to review the guidelines and set the timeline to 39 weeks. Obstetricians usually wait till 41 weeks for spontaneous labour and usually encounter foetal distress with meconium-stained liquor.^9^

Recently in the year 2020 Grenoble classification is introduced considering the Robson Classification of CS. This tool has classified induction of labour (IoL) in a way that rates of IoL can be calculated in hospitals easily. IoL being the most common procedure, carried out in the labour room, should be classified to justify its indication. Rates of IoL are 20-25% in developed countries mostly accounting for high-risk pregnancies while in some institutions it is around 45%.^10^ Data on IoL in Pakistan is limited.

In our study, Primigravida’s without any risk factors were chosen as normal labor is a challenge in the first pregnancy. Secondly, primigravida is a group in which CS should be avoidable until deemed necessary. An important aspect is the counselling of patients for induction of labour which includes which should be justified according to recent evidence like the shorter first stage of labour.^11^ Similarly, patients may give consent due to foetal concerns such as macrosomia. Patients’ prospective and consent were more toward IoL in a survey carried out in the ARRIVE trial.

### Rationale:

Induction of labour at 39 weeks is safe in healthy women and their babies, provided the baby’s gestational age is confirmed to be at least 39 weeks or older. Inducing labour at 39 weeks would also prevent macrosomia and assisted vaginal delivery. It would also reduce the risk of CS, preeclampsia, gestational hypertension, and stillbirth compared with waiting for conventional induction. This study will the first in this region and will help create awareness and build confidence in obstetrician for early induction of labour.

## METHODS

This open random allocation study was conducted at Gynae/Obst Department JPMC during period form 1^st^ June 2022 to 30^th^ September 2023. Pregnant women attending the anti-natal clinic were informed of the trail were offered to participate. Written consent of both women and husband was taken. Non-probability convenience sampling method was used for induction.

.All singleton primiparous women without risk factors with gestational age 38 weeks 0 days to 38 weeks six days with vertex presentation were randomized using random number table into one of the two groups, Group-A in which induction was done at 39 weeks while in Group-B induction was done conventionally. Women associated with risk factors, e.g., hypertension, diabetes, severe obesity, precious pregnancy, IUGR, foetal anomalies and women in labour or with premature rupture of membranes were excluded.

### Ethical Approval:

IRB approval was taken from the department vide their office letter number F.2-81/2022-GENL/177/JPMC dated 25-05-2023

Gestational age was estimated via LMP and confirmed by the first-trimester scan. On admission, bishop scoring was done, and patients were induced according to their bishop score done by attending Fellow. Four methods were used, Intracervical Foleys with Prostaglandin E2 (PGE2), Intracervical Foleys only, PGE2 only, and Sweep and Stretch. These methods were accompanied by injection Oxytocin (5 to 10 IU) according to the requirement. Mean age ±SD, gestational age and delivery time of included women was calculated and compared between two groups by Student’s t test. Frequency of CS in both groups was compared by χ^2^ test. Frequency of perinatal and neonatal outcomes was also compared between two groups by χ^2^ test.

## RESULTS

Eighty-two women were inducted in Group-A and eighty-five in Group-B. The mean age was 24.57±3.54 years and 24.65 ±3.33 years in Group-A & B respectively (*p* = .890). Gestational age between two groups was significantly different, in Group-A it was 38.24±0.46 weeks and Group-B it was 39.68±0.68 weeks (*p*<.001).The mean delivery time in Group-A was significantly more at 8.12±2.77 hours while in Group-B was 7.0±2.62 hours (*p* = .005). Details are given in Table-I.

**Table-I:** Comparison of Maternal & Gestational Age, Baby Weight, and Labour Times between two groups by Student’s t-test.

	Group-A	Group-B	Sig.

	Mean ±SD	Mean ±SD	
Maternal Age	24.57 ±3.54	24.65 ±3.33	.890
Gestational Age (weeks)	38 ±0	40 ±1.0	<.001*
Baby Wt. (Kg)	3.0 ±0.4	2.8 ±0.7	.061
Membrane Rupture to Delivery Time (hours)	3.22 ±2.08	2.56 ±1.93	.036*
1st Stage Duration (hours)	7.24 ±2.72	5.99 ±2.52	.002*
2nd Stage Duration (hours)	.95 ±.44	.95 ±.49	.978
3rd Stage Duration (minutes)	9.95 ±4.31	10.86 ±4.49	.185
Delivery Time (hours)	8.18 2.77	7.00 ±2.62	.005*

* = Significance ≤.05.

Bishop score was significantly better in the Group-B, poor Bishop scorein Group-A was seen in 65.1% while only in 34.9% in Group-B (*p* = .015). Significant differences in frequencies were found in induction methods, foetal outcomes, baby weight & APGHAR Scores between two groups details are given in Table-II. Frequency of CS between two groups was not statistically significant, it was 5 (6.1%) in Group-A and 2 (2.4%) in Group-B (*p* = .412). Details are given in Table-I. In Group-B, 40 women had spontaneous labour while 45 needed interventions as they reached 40 weeks. In our set up we do not leave women after 40 weeks as it increases the risk of meconium stain liquor.^9^ Foleys & Prostin both were used for induction in 65.7% & 34.3% in Groups A & B respectively. Liquor was clear in 49.1% in Group-A while it was clear in 50.9% in Group-B. No significant difference in frequency of perineal tear was seen, it was 7.32% in Group-A and 9.41% in Group-B (*p* = .625). Also, no significant difference in frequency of NICU admission was seen, in Group-A 8.54% babies were admitted to NICU while in Group-B 16.47% were admitted to NICU (*p* = .122).

**Table-II:** Comparison of Foetal and Maternal Factors between Two Groups by χ^2^ test.

		Group-A	Group-B	Sig.

		N (%)	N (%)	
Bishop	Good	54 (65.85)	70 (82.35)	.015*
	Poor	28 (34.15)	15 (17.65)	
Induced	Spontaneous Labour	0 (0)	40 (47.06)	<.001*
	Foleys & Prostin	67 (81.71)	35 (41.18)	
	Prostin only	3 (3.66)	2 (2.35)	
	Foleys only	8 (9.76)	3 (3.53)	
	Sweep & Strech	4 (4.88)	5 (5.88)	
Liquor	Clear	79 (96.34)	82 (96.47)	.548
	Meconium	2 (2.44)	3 (3.53)	
	Blood Stained	1 (1.22)	0 (0)	
Mode of Delivery	SVD	76 (92.68)	80 (94.12)	.227
	Caesarean	5 (6.1)	2 (2.35)	
	Instrumental	1 (1.22)	3 (3.53)	
Foetal Outcome	Alive with Good APGAR	82 (100)	83 (97.65)	.026*
	Alive with Poor APGAR	0 (0)	2 (2.35)	
	Still Birth	0 (0)	0 (0)	
	NND	0 (0)	0 (0)	
Baby Weight Groups	2.5 to 3.5	77 (93.9)	79 (92.94)	.022*
	less than 2.5	0 (0)	5 (5.88)	
	more than 3.5	5 (6.1)	1 (1.18)	
APGHAR Score	< 5	0 (0)	2 (2.35)	.026*
	5-7	18 (21.95)	32 (37.65)	
	> 7	64 (78.05)	51 (60)	
Perineal Tear	No	76 (92.68)	77 (90.59)	.625
	Yes	6 (7.32)	8 (9.41)	
Indication LSCS	None	77 (93.9)	83 (97.65)	.227
	Foetal Distress	5 (6.1)	2 (2.35)	
	NPOL	0 (0)	0 (0)	
	Malposition	0 (0)	0 (0)	
	Cord Prolapse	0 (0)	0 (0)	
	Malpresentation	0 (0)	0 (0)	
	Other	0 (0)	0 (0)	
NICU Admission	No	75 (91.46)	71 (83.53)	.122
	Yes	7 (8.54)	14 (16.47)	
Mode of Delivery	Vaginal	77 (93.9)	83 (97.65)	.227
	Caesarean	5 (6.1)	2 (2.35)	

## DISCUSSION

After publication of ARRIVE Trial in 2020, many centers all over the world have started implementing it for benefit of the pregnant women and IOL in low risk women with ≥ 39 weeks gestation increased from 30.2% to 36.1% in USA.^12^,^13^ Similar findings were also reported in another study were IOL in ≥ 39 weeks increased from 33.9% to 45.8%.^13^Our study showed lower neonatal risk in intervention group with lesser NICU admission. Maternal caesarean section rates did not show any statistically significant difference among the two groups. These findings are in agreement with those by ARRIVE Trial which showed no difference in terms of perinatal outcome and maternal outcomes in both groups.^12^ The ARRIVE Trial was one of the largest trials conducted but it failed to universally implicate its findings of elective induction at ≥ 39 weeks but external validity of ARRIVE Trial was low.^14^

Sinkey et al conducted a large study on100,000, which also showed better neonatal and maternal outcomes in the induction group were better in the induction group.^2^,^4^ These observations were counter argued in a meta-analysis by Grobman WA et al. in there randomized trial ,who suggested to have lower risk of caesarean section with a ratio of 1: 28.^15^Findings of our study in terms of maternal caesarean section rate were different from these large trials, our study is in agreement with earlier studies that showed increased caesarean section in the induction group.^16^

The caesarean section in our study were mainly due to foetal reasons rather maternal. This finding is supported by a large study conducted in African and Asian women which showed increased rates of foetal distress.^17^ Our findings were similar to the study of Ehrenthal DB et al showed decrease NICU admission and still birth rate.^18^ Many retrospective studies have shown a rise in CS rates on elective induction of labour.^19^ However, a large analysis of 2,860,942 births comparing the CS rates in pre and post ARRIVE era, did not show any increase in CS.^20^ Primary postpartum haemorrhage was not seen in our study in any patient as we follow active management of third stage of labour. The elective induction Group-A results in financial burden to the patient and overall to the country.^21^ Pakistan being low socioeconomic country limits the idea of elective induction.

It also seemed hard to convince the patient on a poor Bishop score in intervention group to admit and induce, when data favoring positive effects of elective induction is still conflicting. Recently WHO labour guide^22^ considers patients 5cm dilated as in active labour in order to decrease the caesarean section rate. Labour care guide focuses on avoiding any intervention before patients are 5cm.The decision of not even admitting a patient before 5cm was made to avoid any unnecessary interventions in women in labour. Induction is associated with PPH, more blood loss, peripartum hysterectomies and maternal complications. PPH was not seen in any patient in our study as they all were low risk. Perineal tears were eight in non-intervention Group-And six in intervention group. There wasn’t much difference in both groups.

The first stage of labour was eight hours in intervention Group-A and seven hours in the non-intervention group. There wasn’t much difference in both groups. Although if we compare the cost, intervention group was more costly than spontaneous group. The duration of stay of induction group is more as compared to non-intervention group.

### Limitations:

This is a single centre study with adequate sample size to study the difference between the two groups. Although the internal validity of this study was adequate, external validity of this study was low as the extent to which the study population and setting are representative of the larger source population the study intends to represent is low.

## CONCLUSIONS

Our study showed non-significant increase in CS in women who were induced at 39 weeks, while no significant difference was observed in foetal, neonatal and maternal outcomes.

### Authors Contribution:

**EM:** Conceived the idea, Final approval of manuscript, responsible of the integrity of research.

**BFZ:** Statistical analysis and manuscript writing.

**KH:** Data collection and entering data in SPSS.

**HJ and GH:** Draft manuscript writing, data Collection.
